# Radiosensitizing Pancreatic Cancer with PARP Inhibitor and Gemcitabine: An In Vivo and a Whole-Transcriptome Analysis after Proton or Photon Irradiation

**DOI:** 10.3390/cancers13030527

**Published:** 2021-01-30

**Authors:** Waisse Waissi, Anaïs Nicol, Matthieu Jung, Marc Rousseau, Delphine Jarnet, Georges Noel, Hélène Burckel

**Affiliations:** 1 Centre Leon Bérard, Department of Radiation Oncology, 69008 Lyon, France; waisse.waissi@lyon.unicancer.fr; 2 Paul Strauss Comprehensive Cancer Center, Radiobiology Laboratory, Institut de Cancérologie Strasbourg Europe (ICANS), Strasbourg University, UNICANCER, 67000 Strasbourg, France; a.nicol@icans.eu; 3 Institut de Génétique et Biologie Moléculaire et Cellulaire (IGBMC), UdS, CNRS, INSERM, 1 rue Laurent Fries, B.P. 10142, 67404 Cedex Illkirch, France; jung@igbmc.fr; 4 Institut Pluridisciplinaire Hubert Curien, Strasbourg University, CNRS, UMR 7178, 67200 Strasbourg, France; marc.rousseau@iphc.cnrs.fr (M.R.); g.noel@icans.eu (G.N.); 5 Paul Strauss Comprehensive Cancer Center, Medical Physic Unit, Institut de Cancérologie Strasbourg Europe (ICANS), UNICANCER, 17 Rue Albert Calmette, 67200 Strasbourg, France; d.jarnet@icans.eu; 6 Paul Strauss Comprehensive Cancer Center, Department of Radiation Oncology, Institut de Cancérologie Strasbourg Europe (ICANS), UNICANCER, 17 Rue Albert Calmette, 67200 Strasbourg, France

**Keywords:** pancreatic cancer, proton therapy, radiotherapy, DNA repair, gemcitabine, PARP inhibitor, transcriptome

## Abstract

**Simple Summary:**

Pancreatic ductal adenocarcinoma is a devastating disease. Using modern technique of radiotherapy, such as proton therapy, may simultaneously enhance dose to the tumor and decrease dose to surrounding organ, thus limiting toxicity. Moreover, associating drugs to radiotherapy also increases its effectiveness on tumor. The aim of our study was to show the benefit of proton therapy compared to standard radiotherapy with photon, and the benefit of associating different drugs with those particles in vivo. Thus, our results displayed a higher effectiveness of associating proton therapy, gemcitabine and olaparib. Finally, we pointed out that treatment induced significant transcriptomic alterations.

**Abstract:**

Over the past few years, studies have focused on the development of targeted radiosensitizers such as poly(ADP-ribose) polymerase inhibitors. We performed an in vivo study and a whole-transcriptome analysis to determine whether PARP inhibition enhanced gemcitabine-based chemoradiosensitization of pancreatic cancer xenografts, combined with either proton or photon irradiation. NMRI mice bearing MIA PaCa-2 xenografts were treated with olaparib and/or gemcitabine and irradiated with 10 Gy photon or proton. First, a significant growth inhibition was obtained after 10 Gy proton irradiation compared to 10 Gy photon irradiation (*p* = 0.046). Moreover, the combination of olaparib, gemcitabine and proton therapy significantly sensitized tumor xenografts, compared to gemcitabine (*p* = 0.05), olaparib (*p* = 0.034) or proton therapy (*p* < 0.0001) alone or to the association of olaparib, gemcitabine and radiotherapy (*p* = 0.024). Simultaneously, whole RNA sequencing profiling showed differentially expressed genes implicated in categories such as DNA repair, type I interferon signaling and cell cycle. Moreover, a large amount of lncRNA was dysregulated after proton therapy, gemcitabine and olaparib. This is the first study showing that addition of olaparib to gemcitabine-based chemoradiotherapy improved significantly local control in vivo, especially after proton therapy. RNA sequencing profiling analysis presented dynamic alteration of transcriptome after chemoradiation and identified a classifier of gemcitabine response.

## 1. Introduction

Pancreatic ductal adenocarcinoma (PDAC) is considered as one of the most aggressive cancers [1]. Approximately 30% of patients are diagnosed with a locally advanced disease, reducing the median overall survival (OS) to only 24 months [2]. New approaches emerged to improve the OS of PDAC patients, as demonstrated by two randomized clinical studies that confirmed the superiority of chemoradiotherapy (CRT) over radiotherapy (RT) alone [3]. However, the overall downstaging rate after CRT treatment is only 4–15%. Altogether, a more in-depth interest in the development of novel therapies, such as CRT combinations, needs to be assessed. PDAC patients enrolled in RT protocols are commonly treated with photon therapy, specifically X-rays. This method, despite being precise thanks to the intensity-modulated radiotherapy (IMRT), does not allow radiation dose escalation due to the risk of toxicity in the surrounding healthy tissues. Typically, photon interact with the milieu and generate electrons that deposit their dose locally. Remaining photon continue after the region of interest, here the tumor, and create an unintended dose deposition in healthy tissue in the vicinity of the tumor. With the rise of proton therapy, novel perspectives are expected as they can be better controlled and hence the dose deposition is more targeted. This advantage of proton over photon enables a potential dose escalation [4]. Apart from the ballistic advantage, proton enhance biological effectiveness in cell killing compared to photon, related to the increased linear energy transfer (LET) in the Bragg peak [5,6].

The pancreas is surrounded by highly radiosensitive normal tissue such as duodenum, kidneys or liver; therefore, the use of proton therapy is of great interest for PDAC treatment, to better conform to tumor targets, while sparing normal tissue [7]. RT alone will not cure definitively the tumor due to potential circulating tumor cells, or to some RT resistant tumor cells that may allow further tumor regrowth. Combining this approach with CRT could then be an interesting option. Gemcitabine is widely used as a radiosensitizer for PDAC treatment and is known to induce tumor cells S-phase arrest, which is a cell cycle phase known to sensitize cells to DNA damage, one of the mechanisms of cell death induced by RT [8]. Indeed, cells are usually resistant to irradiation in S-phase due to sister chromatid and the possibility to accurately repair double strand breaks through homologous recombination. However, the situation is different when nucleoside pool is imbalanced and availability of sister chromatid is scarce [9].

On the other hand, poly(ADP-ribose) polymerase-1 (PARP-1) has an essential role in the recognition of DNA damage and initiation of DNA single- and double-strand breaks repair. Therefore, PARP inhibitors could sensitize cells to exogenous DNA damage inducer treatment, such as irradiation or gemcitabine [10]. Hence, the mechanism of radiosensitization with PARP inhibitors is specific of S cell cycle phase, as it involves stalled replication forks [11]. Thus, PARP inhibition could particularly radiosensitize gemcitabine-based CRT. Therefore, in order to boost the efficacy of the combination of gemcitabine and proton therapy, we suggested increasing DNA damage by adding a PARP-1 inhibitor to the association, such as olaparib.

In this study, we sought to determine whether treatment with gemcitabine, combined to proton therapy and reinforced by DNA-damage radiosensitization using a PARP inhibitor, olaparib, is a viable strategy to improve the treatment of PDAC. Moreover, we analyzed transcriptomic alterations to identify the molecular process and pathways involved in response to this multimodal treatment. 

## 2. Results

### 2.1. Olaparib, Gemcitabine and Proton Therapy Significantly Inhibit Tumor Growth In Vivo

**Figure 1:**
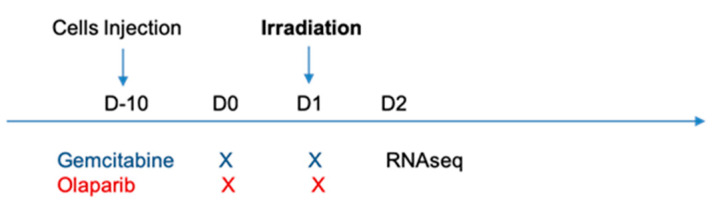
Tumor-bearing mice (*n* = 15 per group) were treated with DMSO, olaparib (50 mg/kg) and/or gemcitabine (40 mg/kg) for two consecutive days. One hour after treatment injection, mice were randomized for 10 Gy irradiation with photon, proton or sham. Five tumors per group were harvested 24 h after irradiation for RNA sequencing analysis. Tumor volumes were evaluated three times a week on 10 tumor-bearing mice per groups.

Mice bearing MIA PaCa-2 xenografts were treated with 50 mg/kg olaparib or 40 mg/kg gemcitabine or both associated treatments for two consecutive days (Figure 1). One hour after the last treatment injection, tumors were locally irradiated with 10 Gy photon (radiotherapy), proton or sham irradiation.

**Figure 2:**
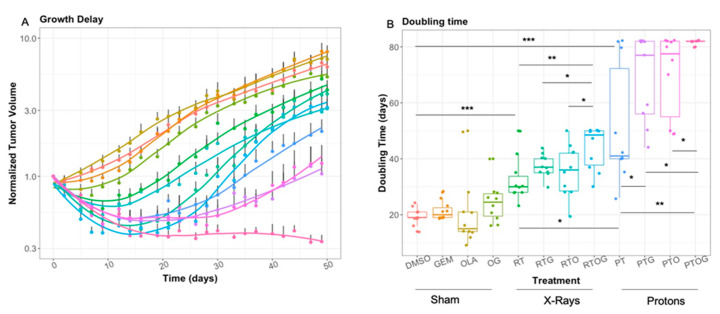
Athymic nude mice bearing subcutaneous MIA PaCa-2 xenografts were treated with DMSO, gemcitabine (GEM), olaparib (OLA), olaparib and gemcitabine (OG), radiotherapy (RT), radiotherapy and gemcitabine (RTG), radiotherapy and olaparib (RTO), radiotherapy, olaparib and gemcitabine (RTOG), proton therapy (PT), proton therapy and gemcitabine (PTG), proton therapy and olaparib (PTO) and proton therapy, olaparib and gemcitabine (PTOG). Mice were followed until 50 days (photon) or 80 days (proton). Mean normalized tumor volume ± SE (**A**) and mean tumor volume doubling time (**B**) are described. Each experimental group contained 10 mice per group. * *p* < 0.05, ** *p* < 0.01, *** *p* < 0.001.

**Figure 3:**
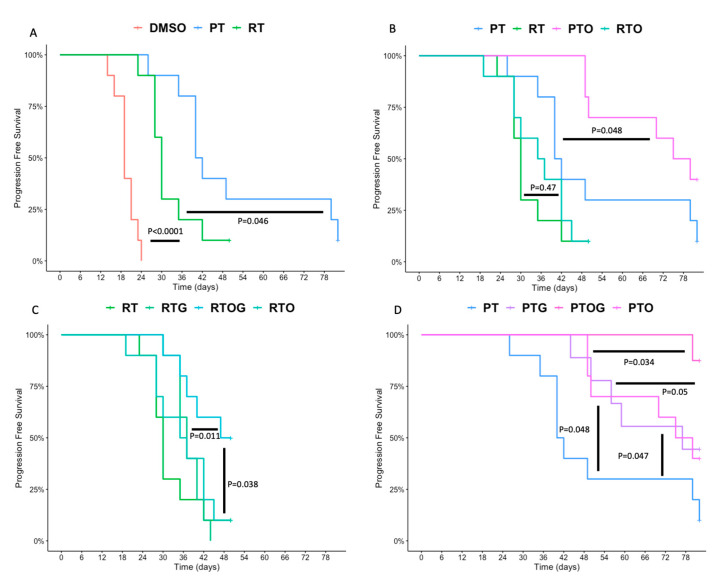
Athymic nude mice bearing subcutaneous MIA PaCa-2 xenografts were treated with (**A**): DMSO (control, red), radiotherapy (10 Gy, blue) or proton (10 Gy, green); (**B**): photon (10 Gy, green) or proton (10 Gy, red), olaparib and photon (purple) and olaparib and proton (blue); (**C**): photon (10 Gy, red) alone, the combinations of olaparib and photon (purple) or gemcitabine and photon (green) and the triple therapy of olaparib, gemcitabine and photon (blue) and (**D**): proton therapy alone (red), the combinations of olaparib and proton therapy (purple) or gemcitabine and proton (green) and the triple therapy of olaparib, gemcitabine and proton (blue). Mice were followed until 50 days (photon) or 80 days (proton). Progression-free survival was designated as the time when tumor doubled compared to the tumor size before treatment (day 0). Each experimental group contained 10 mice per group.

Gemcitabine or olaparib as single agent, or their combination, had no significant effect on tumor growth or tumor doubling time (Figure 2A,B). In contrast, a single dose of 10 Gy photon significantly inhibited growth delay and enhanced tumor-doubling time compared to sham irradiation (*p* < 0.0001, Figure 2 and Figure 3A). Moreover, a single dose of 10 Gy proton irradiation significantly increased tumor growth delay compared to photon irradiation (*p* = 0.046, Figure 2 and Figure 3A). 

Indeed, the median time for tumor to reach twice their initial volume was 19 days for controls (DMSO), 30 days after radiotherapy (photon) and 40 days with proton therapy (Table S1). Thus, enhancement factor was 1.3 with proton compared to photon irradiation, meaning that proton therapy enhanced tumor doubling-time by 30%.

To investigate the potential radiosensitization effect of olaparib, tumor-bearing mice were treated with olaparib 24 h and 1 h before irradiation (Figure 1). While photon alone significantly reduced tumor growth as previously presented, pre-treatment with olaparib did not significantly enhanced progression-free survival (*p* = 0.47) (Figure 3B), nor tumor-doubling time (Figure 2B), compared to radiotherapy alone. However, pre-treatment with olaparib before proton therapy significantly enhanced progression-free survival and tumor-doubling time, compared to proton therapy alone (*p* = 0.048) (Figure 3B). Thus, olaparib was an effective radiosensitizer on PDAC tumor xenografts with proton therapy, whereas no significant effects were found with radiotherapy. 

**Figure 4:**
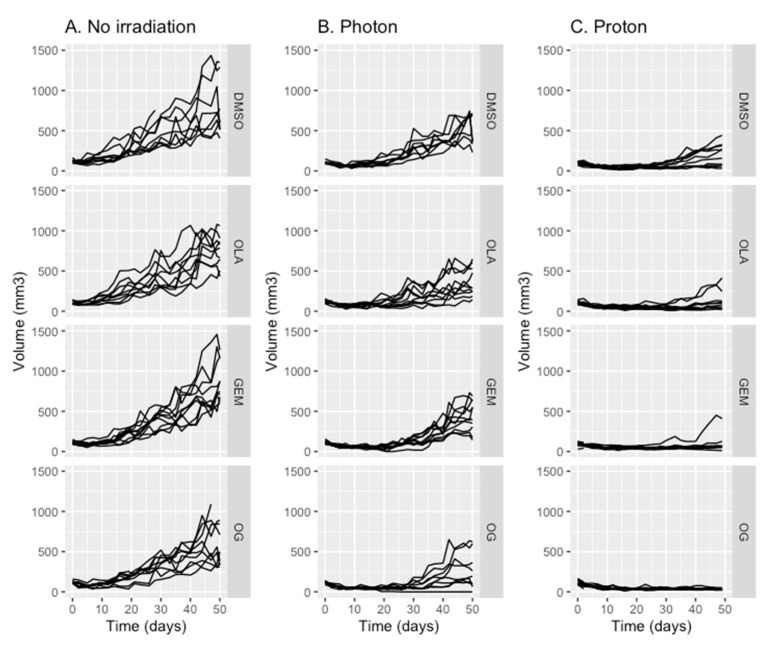
Growth (volume in mm^3^) of MIA PaCa-2 tumors in athymic nude mice treated with DMSO (control), olaparib (OLA), gemcitabine (GEM) or olaparib and gemcitabine (OG) with either sham irradiation (no irradiation) (**A**), radiotherapy (**B**) or proton therapy (**C**). Each experimental group contained 10 mice per group.

As gemcitabine-based chemoradiotherapy is a treatment option for locally advanced PDAC, we evaluated whether olaparib increased tumor response to the combination of gemcitabine and irradiation (Figure 2 and Figure 4A–C). Olaparib sensitized PDAC tumor xenografts to gemcitabine-based chemoradiation, as evidenced by significantly higher median tumor-doubling time in response to triple combination compared to the associations of gemcitabine and irradiation or olaparib and irradiation (Figure 2). Although pre-treatment with olaparib significantly enhanced tumor growth delay after photon (*p* = 0.0011) or proton gemcitabine-based chemoradiotherapy (*p* = 0.05), proton radiosensitization was more effective compared to photon (*p* = 0.024) (Figure 3C,D).

Finally, after 50 days, 90% of tumor-bearing mice treated with the triple combination of olaparib, gemcitabine and proton therapy had objective responses, defined as the decrease of two-fold of their initial volume for 6 mice and even absence of tumor (complete response) for three of them (Figure 4C). In contrast, the objective response rate with radiotherapy-based triple combination was only 10% after 50 days follow-up (Figure 4B).

### 2.2. RNA Sequencing Identifies Pathways Involved in Radiosenstization

Gene transcripts were analyzed 24 h after irradiation in each group. The number of transcripts significantly differentially expressed (DE) was determined according to treatment comparisons (Table 1).

To identify possible genes and pathways affected by radiosensitization with gemcitabine, olaparib or the combined treatments, RNA sequencing profiling was performed on excised MIA PaCa-2 xenografts models. The numbers of DE transcripts between all contrasts are reported in Table 1. The most relevant differentially expressed transcripts were encountered between proton therapy, olaparib and gemcitabine (PTOG) comparison and proton therapy (PT) alone, with 1 679 DE transcripts. Then, second most relevant differences were observed between DMSO control (CTL) and PT, with 778 DE transcripts. Finally, we also observed 657 DE transcripts between the contrast of proton therapy and olaparib (PTO) and PT alone.

**Figure 5:**
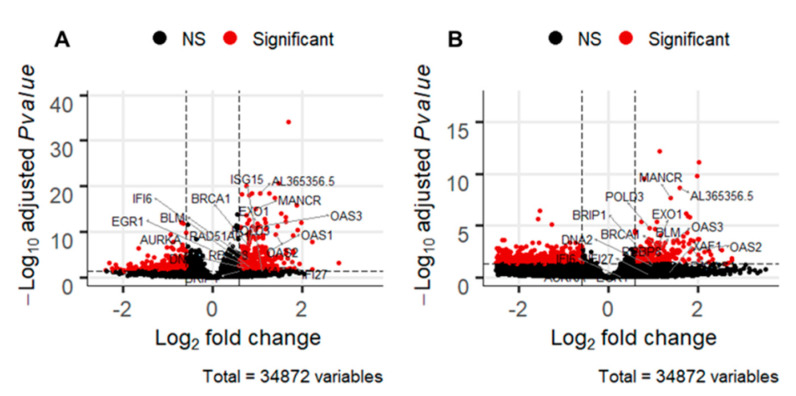
Volcano plot illustrating log2 fold change compared with *p* value (−log base 10) between (**A**) tumors treated with or without gemcitabine and (**B**) proton therapy (PT) vs. proton therapy, olaparib and gemcitabine (PTOG). Horizontal bars represent a significance level of *p* = 0.05 and vertical bars represent a significant log2 fold change. The red points represent the transcripts with a fold change ≥ 1.5 and a *p*-value ≤ 0.05. The 10 most differentially expressed transcripts in gemcitabine vs. no gemcitabine contrast are labeled in the black boxes, among them, the most significantly dysregulated lncRNAs. Black dot: non-significant; red dot: significant.

Besides this, our whole RNA sequencing profiling also revealed an interesting amount of lncRNA (Table 1). We found that two lncRNA (*MANCR* and *AL365356.5*) were differentially expressed in five comparisons (RTOG vs. RT, RTO vs. RTOG, PT vs. PTG, PTOG vs. PT, PTO vs. PTOG). Moreover, when we compared all conditions with gemcitabine and without gemcitabine, we found that these lncRNA were underexpressed in gemcitabine conditions (*MANCR*: logFC = 0.957 and AdjPval = 8.32 × 10^−16^; *AL365356.5*: logFC = 1.05 and AdjPval = 4.46 × 10^−19^) (Figure 5).

**Figure 6:**
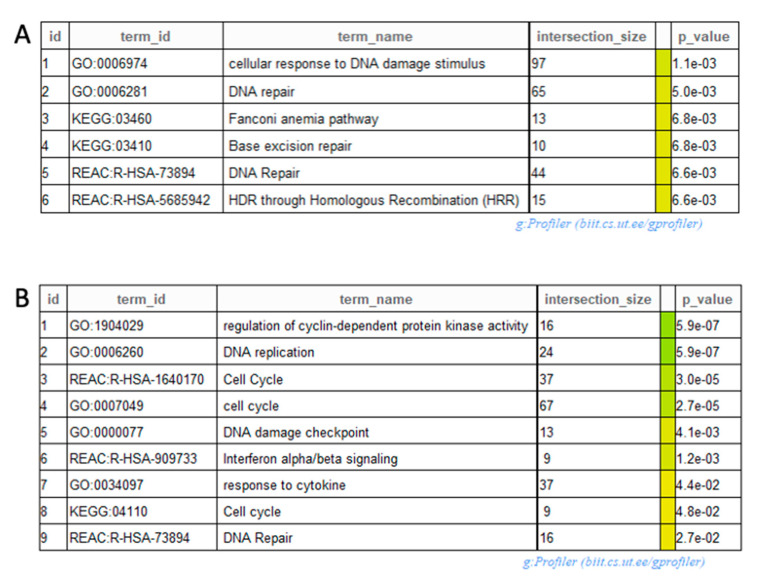
Relevant categories obtained after enrichment (**A**) for proton therapy, olaparib and gemcitabine (PTOG) vs. proton therapy (PT) alone and (**B**) for gemcitabine vs. no gemcitabine treatments.

Enrichment analyses were performed for all comparisons. In the most relevant comparison (PTOG vs. PT) DE genes involved in DNA repair such as “Base excision repair,” “Fanconi Anemia pathway” and “HDR through Homologous recombination repair” were found to be significantly associated in response to triple association compared to proton therapy alone (Figure 6A). Based on the observation that gemcitabine dysregulated lncRNA, we evaluated the consequences of gemcitabine treatment. Enrichment analysis displayed that DE genes implicated in biological categories such as “DNA repair,” “type I interferon signaling” and “cell cycle” were significantly associated in response to gemcitabine treatment (Figure 6B).

**Figure 7:**
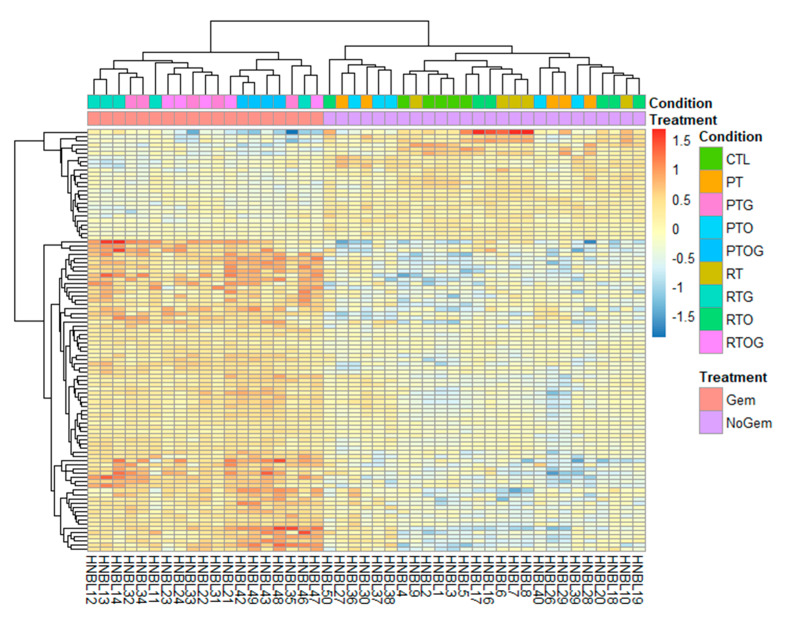
Heatmap presenting the 100 more significant gene expression levels in RNA samples of MIA PaCa-2 tumors treated with (Gem, pink) or without gemcitabine (NoGem, purple). Treatments are defined as DMSO (CTL), radiotherapy (RT), radiotherapy and gemcitabine (RTG), radiotherapy and olaparib (RTO), radiotherapy, olaparib and gemcitabine (RTOG), proton therapy (PT), proton therapy and gemcitabine (PTG), proton therapy and olaparib (PTO) and proton therapy, olaparib and gemcitabine (PTOG). Five samples were evaluated per condition.

Then, using supervised clustering analysis, two clusters were found representing tumors treated with gemcitabine-based irradiation and tumors that were not treated with gemcitabine, irrespective of the type of irradiation or the treatment with olaparib. We identified a 100-transcript signature highly correlated with gemcitabine treatment (Figure 7). Among these transcripts, there was a strong negative association between tumors treated with gemcitabine and expression of the AURKA gene. The TCGA database was assessed and we determined that patients with under-expression of *AURKA* had a significant better overall survival (Figure S1). This is consistent with our data exhibiting that downregulation of *AURKA* after gemcitabine-based CRT could enhance tumor response.

## 3. Discussion

In this study, we investigated the potential of olaparib, a potent PARP inhibitor, to radiosensitize PDAC xenografts after gemcitabine-based chemoradiotherapy with radiotherapy or proton therapy. This tumor growth study highlighted that proton therapy alone significantly increased progression-free survival, compared to photon radiotherapy. It is well known that the relative biologic effectiveness (RBE) is defined as the ratio between the dose delivered in photon and proton irradiation, achieving the same specified biologic effect [12]. As recommended by the ICRU 78, it is common to use a constant generic RBE of 1.1 in clinical studies [13]. However, in our study, 10 Gy proton irradiation delayed tumor growth by enhancing tumor-doubling by 30%, compared to photon with the same physical dose and dose rate. Therefore, we could assume that RBE would be 1.3 with MIA PaCa-2 PDAC xenografts. Indeed, the RBE for proton therapy is considered as a complex function of cell type α/β, LET, dose and endpoint [12]. As the 1.1 RBE is a general definition, in this particular case of PDAC tumors, the difference of effect observed between photon and proton irradiation could be due to previous cited factors. Finally, the limit of 6 mm for the tumor depth could be at the end of the SOBP, explaining partially a relatively higher RBE than expected.

In our study, olaparib did not radiosensitize pancreatic cancer xenografts after photon irradiation. This was consistent with Karnak et al., who evaluated fractionated radiotherapy (photon) with olaparib in MIA PaCa-2 xenografts model and determined that olaparib did not induce any radiosensitization [14]. Other publications displayed no radiosensitization with olaparib in PDX model, in BRCA-WT PDAC tumors [15]. In contrast, in our study, olaparib radiosensitized PDAC MIA PaCa-2 xenografts treated with proton therapy. Recently, Hirai et al. determined that treatment of MIA PaCa-2 cell line by olaparib before proton irradiation enhanced radiosensitization, specifically in the SOBP region, compared to the entrance region, thus demonstrating that PARP inhibition radiosensitized cancer cells in a LET-dependent manner [16]. The major difference between photon and proton irradiation lies in the LET. Indeed, the average LET of conventional megavoltage radiotherapy is around 0.2 keV/μm, whereas in the spread-out Bragg peak region, the simulated LET was between 2–3 keV/μm, which is approximately ten times higher than megavoltage photon [12]. DNA damages induced by high LET particles, such as proton, are more complex and clustered than those induced by photon [17], particularly closely associated oxidized base and single-strand breaks. These lesions are mainly repaired by base excision repair, in which PARP plays a significant and predominant role [18,19]. 

Furthermore, we displayed that the associations of gemcitabine and olaparib relevantly enhanced gemcitabine-based chemoradiotherapy with both photon and proton irradiation. Only two studies investigated proton-based radiosensitization with PARP inhibitors and both of them were in vitro studies [16,20]. To the best of our knowledge, this is the first study evaluating proton-based chemoradiosensitization with gemcitabine and olaparib in a preclinical in vivo model of PDAC xenografts. Moreover, some studies evaluated other DNA damage response inhibitors to sensitize gemcitabine-based chemoradiation, in preclinical model of PDAC xenografts [21,22,23]. Indeed, Kausar et al. evaluated AZD1775, a Wee1 inhibitor, in PDAC PDX model and presented enhancement of tumor-doubling time with association of photon radiotherapy, gemcitabine and AZD1775, compared to gemcitabine radiotherapy [21]. Fokas et al. evaluated the potential role of an ATR inhibitor, VE-822, to sensitize PDAC xenografts to gemcitabine-based chemoradiotherapy. The addition of VE-822 to the combination of gemcitabine and photon irradiation extended tumor growth delay, compared to radiotherapy and gemcitabine [23]. Engelke et al. determined that MIA PaCa-2 xenografts were significantly sensitized to gemcitabine-based chemoradiation by Chk1 inhibitor [22]. All these data highlighted that targeting DNA damage response pathways could be effective to enhance gemcitabine-based chemoradiotherapy, thus translating into better clinical outcomes. Recently, Görte et al. evaluated phosphoproteomics changes after photon and proton irradiation and showed that proton therapy stimulates greater phosphoproteome changes compared to photon. Moreover, targeting classical and alternative NHEJ enhanced therapeutic ratio either with photon or proton [24]. 

Tuli et al. published results of a phase I clinical trial assessing efficacy of gemcitabine, fractionated IMRT and veliparib, a PARP inhibitor, in locally advanced PDAC [25]. Authors emphasized that co-treatment with veliparib and gemcitabine was well tolerated and median progression-free survival was 9.8 months (95% CI: 8.4–18.6). Moreover, baseline poly(ADP-ribose) levels, tumor mutational burden or microsatellite instability were not correlated with survival. Thus, assessing that transcriptional response to treatments could help to better identify biomarkers of response. 

We assessed tolerance of the triple association by evaluation the weights of all mice 3 times per week and no statistically significant weight losses were observed between the day of irradiation and 10 days after, for all treatment combinations. However, a transitory weight loss was observed 7 days post-irradiation when mice were treated with the combination of olaparib and gemcitabine (with or without irradiation) with no symptom at the physical examination, but they all recovered their normal weight one week later. In a recent phase I study, the combination of gemcitabine, photon irradiation and veliparib (PARPi) has been assessed [25]. The major grade 3–4 toxicity was hematological and principally due to Veliparib. It is known that associating radiotherapy and systemic treatment is a major issue. As the dose at the organ at risk is significantly lower, association of systemic treatment with proton therapy could significantly reduce toxicity compare to radiotherapy.

Bioinformatics analyses were performed to investigate transcriptional response after irradiation, associated or not with gemcitabine and/or olaparib. First, transcriptomic responses were affected in various ways by all treatment’s associations. Indeed, a wide range of DE transcripts between different conditions varied from no to maximum 1679 significantly DE transcripts. This emphasized that irradiation and/or their combination with olaparib and/or gemcitabine could sometimes highly altered early transcriptomic responses. The number of DE transcripts was important in PTOG vs. PT comparison, and meaningful dysregulated lncRNA were identified. LncRNAs are newly recognized as regulators of genes expressions, transcriptionally and post-transcriptionally, thus affecting mRNA biogenesis [26]. In our study, by means of an in-depth analysis, many long non-coding RNAs were found differentially expressed between some conditions with irradiation. Indeed, an important number of lncRNAs have been identified to be part of intercellular communication, and could provide drug resistance [27]. Thus, lncRNAs could be used as a diagnostic tool or biomarkers of response which could be easily assessed with fluid sample through liquid biopsy. Gene Ontology and Kegg pathway enrichment analyses are major processes for investigating gene group that contribute in common biological processes or molecular functions. However, genes affected by lncRNAs are not considered in these approaches. There have been lots of evidence that lncRNAs may affect the number of biological processes of cancer cells, such as proliferation, cell cycle regulation, DNA repair, cell death, invasion and metastasis [28]. However, less is known about the potential prognosis value of lncRNAs, particularly after irradiation. In our analysis, we also identified two important lncRNAs involved in response to gemcitabine: *MANCR* (Mitotically Associated Long Non Coding RNA) and *AL365356.5*. *MANCR* has recently been identified as a major component of cellular proliferation, and migration [29,30,31]. Moreover, upregulation of *MANCR* could predict poor prognosis in patients with gastric cancer [32]. However, it has never been identified as a predictive factor of response after treatment, particularly in pancreatic cancer.

Understanding the transcriptomic response after antineoplastic agents’ treatment is a matter of concern. Thus, we aimed to identify biological process involved in gemcitabine response. Enrichment analysis displayed that DE genes implicated in biological process such as “DNA repair”, “type I interferon signaling” and “cell cycle” were significantly involved in response to gemcitabine. Gemcitabine is a deoxycytidine analog interfering with DNA replication, explaining the transcriptomic response of biological process involved in DNA synthesis and DNA repair. Less is known about immunomodulatory effects of gemcitabine in PDAC cells [33].

Knowing that PDAC microenvironment is mostly composed of extremely immunosuppressive cells such as T regulatory cells (Treg), tumor associated macrophages (TAMs) and myeloid derived suppressive cells (MDSCs), it explains resistance to immune checkpoint inhibitors [34]. Recently, it has been presented that irradiation and PARP inhibitor could enhance immune response [35]. Indeed, PARP inhibitors and radiation can upregulate the expression and secretion of chemokines such as *CCL2*, *CCL5*, *CXCL16* and *CXCL10* [35]. Thus, adding PARP inhibitor to irradiation could reverse intrinsic microenvironment immunosuppressive state and sensitize PDAC to immune checkpoint inhibitors. Then, it could be valuable to evaluate tumor microenvironment and the immune response to radiation with DNA damage response inhibitor association, in models such as genetically engineered mouse model (GEMM) [36,37]. 

Finally, using supervised clustering, our analysis identified a gemcitabine response signature. Indeed, we displayed a high correlation between under-expression of AURKA and gemcitabine treatment. The Aurora kinases comprise a family of three homologs serine/threonine kinases that play an essential role in cell cycle progression, particularly in G2/M phase. Among them, Aurora kinase A participates in centrosome assembly and is important for the maintenance of genomic integrity. It is well known that overexpression of *AURKA* is associated with tumor proliferation and chromosomal instability [38]. Thus, downregulation of *AURKA* after treatment with gemcitabine-based CRT could explain the observed responses in our study and is coherent with TCGA database. 

## 4. Materials and Methods 

### 4.1. Cell Line and Mouse Model

All procedures were assessed under protocols approved by the French Ministry of Agriculture APAFIS#14091-2018031512594332 v1 and APAFIS#11951-201706091022756 v4, in accordance with the ethical rules for the care and use of animals for research. 

MIA PaCa-2 cells (5.106) were suspended in 1:1 mixture of 10% fetal bovine serum/DMEM:Matrigel and injected subcutaneously in the right flank of 5-week, athymic, female NMRI-Foxn1 nu/nu mice (Janvier Labs, Saint Berthevin, France). Ten days after injection, mice were randomized in different treatment groups to obtain an equivalent tumor volume average in each group of 100 ± 20 mm^3^ (*n* = 10 for growth delay study and *n* = 5 for RNA-Seq profiling analysis). Range of tumor starting volume have been tested not to be different for all evaluated groups (*p* = 0.069). In preliminary data we assessed the depth of various tumors ten days after injection of the cells and all tumors were under 6 mm depth. We had two cohorts as the proton and photon irradiation facilities are not at the same location, but both cohorts had their own control group and no difference was observed between the control group in each facility. Based on tumor doubling time, we did a post hoc Mann-Whitney test with 10 animals per group and we found a power of 72% between photon and proton groups.

Tumor size was measured three times a week using a caliper until tumor volume reached the limit point of 1500 mm^3^. Tumor volume (TV) was calculated according to the equation: TV = (L × W^2^)/2, where L and W are the length and width dimensions of the tumor, respectively. Mice were euthanized as soon as a limit point was reached for the growth delay study; for RNA-Seq experiments, mice were sacrificed 24 h after irradiation. Survival times were calculated from the day of randomization to death (TV ≥ 1500 mm^3^).

### 4.2. Treatment Protocols

Gemcitabine (40 mg/kg) and olaparib (50 mg/kg) were administered via intra-peritoneal injections 24 h and 1 h before irradiation, respectively (Figure 1). Gemcitabine (Selleck Chemicals LLC, Houston, TX, USA) was dissolved in dimethyl sulfoxide (DMSO) and diluted in PBS at 40 mg/mL. Olaparib (Selleck Chemicals LLC) was dissolved in DMSO at a concentration of 50 mg/mL then diluted in PBS and 10% 2-hydroxypropyl-β-cyclodextrin (Sigma, St. Louis, MO, USA) at 4 mg/mL.

### 4.3. Irradiation Protocols

Mice were irradiated with either clinical photon beams or clinical proton beams under anaesthesia (ketamine 100 mg/kg and xylazine 10 mg/kg). Tumors were irradiated with a single fraction of 10 Gy of physical dose and a dose rate of 8 Gy/min. 

#### 4.3.1. Photon Irradiation

Photon irradiation were carried out using a Novalis Tx (Varian Medical Systems, Palo Alto, CA, USA), with 6 MV energy photon in Paul Strauss Comprehensive Cancer Center (ICANS, Strasbourg, France). To plan dose irradiation, an ionization chamber (Pinpoint 0.016 cc, PTW, Freiburg) was introduced in a water equivalent material corresponding to the center of the irradiated tissue volume. This phantom material was irradiated in the same experimental conditions as mice to optimize the delivery of the 10 Gy irradiation dose in all the tumor volume. The tumor was immobilized and its surface was covered with 1 cm bolus to ensure the build-up and dose homogeneity in the tumor thickness. The dose of 10 Gy was homogeneously delivered with a single beam at 90°. The whole body of the mouse, apart from the tumor, was protected with lied shield (XRaystore, La Garde, France) to avoid radiation toxicity. 

#### 4.3.2. Proton Irradiation

Proton beam was extracted from the accelerator of the Cyrcé platform (CYclotron pour la ReCherche et l’Enseignement) in Institut Pluridisciplinaire Hubert Curien (Strasbourg, France) with an averaged energy beam of 25 MeV. Using an in-house immobilization bed, tumor was directly irradiated in contact with a 10 mm diameter collimator. A scattering spread-out Bragg peak (SOBP) field was calculated from an analytical algorithm based on PStar Databased [39]. A beam energy degrader wheel was then used to produce and deliver the SOBP at a maximum depth of 6 mm. Further information on dosimetry, characterization of the beam and dose-averaged linear energy transfer over the SOBP could be obtained in reference [40].

### 4.4. RNA Extraction, RNA-Seq Profiling and Gene Enrichment Analysis

Animals were euthanized 24 h after irradiation by either proton or photon beams. Tumor was then harvested, flash frozen in liquid nitrogen and stored at −80 °C. Total RNA was extracted using RNeasy Plus Universal Tissue Mini kit (Qiagen, Hilden, Germany) as per manufacturer’s instructions. RNA integrity was performed using an Agilent 2100 Bioanalyzer (Agilent Technologies, Palo Alto, CA, USA). RNA quantification and quality (ratio of OD260/230 and OD260/280) were assessed using a NanoDrop 1000 Spectrophotometer (Thermo Fisher Scientific, Wilmington, DE, USA).

The RNA sequencing procedure was performed by the GenomEast platform, Institut de Génétique et de Biologie Moléculaire et Cellulaire (Illkirch, France), a member of the ‘France Genomic consortium’ (ANR-10-INBS-0009). RNA-Seq libraries were generated from total RNA using TruSeq Stranded mRNA LT Sample Preparation Kit (Illumina, San Diego, CA, USA), according to manufacturer’s instructions. Once qualified, single-end libraries were sequenced using 2 × 50 bp output on a HiSeq 4000 device (Illumina).

Resulting reads were processed using an in-house RNA-Seq pipeline of GenomEast facility. Briefly, cutadapt version 1.10 was used for reads preprocessing: trimming of adapter and low-quality (Phred quality score below 20) bases and removal reads shorter than 40 bp after trimming. Reads mapping to rRNA and spike sequences were also discarded. Remaining reads were then mapped onto a hybrid genome composed of hg38 assembly of Homo sapiens and mm10 of Mus musculus genomes using STAR version 2.5.3a [41]. Gene expression was quantified using htseq-count release 0.6.1p1 with “union” mode and gene annotations from Ensembl release 93 [42]. Data have then been split in order to only keep human read counts for further analysis.

RNA-Seq profiling analysis was performed with the free software R (R version 3.6.0). The DESeq2 package (version 1.24.0) was used to execute differential analysis between all treatment modalities [43]. Genes with an adjusted *p*-value ≤ 0.05 and a log2 fold change ≥ 0.058 were considered differentially expressed (DE) and represented in a volcano plot, obtained with the package EnhancedVolcano [44]. To identify biological categories within each cluster, annotations and enrichment analyses of the DE genes were conducted using gProfiler2 v0.2.0. [45,46].

### 4.5. Statistical Analysis

The results were expressed as mean ± standard error (SE). All figures and statistical analysis were assessed using R software (R version 3.6.0). A *p*-value ≤ 0.05 was considered to be statistically significant. For tumor growth experiments, the time required for tumor volume doubling was determined for each xenograft by identifying the earliest day by which the volume was twice higher than before treatment. Kaplan-Meier curves were constructed for analysis of doubling time and log-rank test was performed to compare doubling time between two treatment groups.

## 5. Conclusions

To the best of our knowledge, this is the first study evaluating tumor growth and transcriptional changes after treatment of PDAC xenografts mice model with the combination of olaparib, gemcitabine and irradiation with either proton therapy or radiotherapy. Our analysis presented that the association of gemcitabine, olaparib and proton therapy significantly enhanced tumor response and progression-free survival in a heterotopic xenografts’ mice model. Finally, the transcriptomic data generated in this study may inspire new studies leading to a better comprehension of dynamic transcriptomic response of radiosensitization with either proton or photon irradiation.

## Figures and Tables

**Table 1:** Numbers of significantly differentially expressed (DE) transcripts according to all treatment comparisons (*p*-value adjusted ≤ 0.05 and |log2FC| ≥ 0.058). Treatment are defined as CTL: control (DMSO); RT: radiotherapy; RTG: radiotherapy and gemcitabine; RTO: radiotherapy and olaparib; RTOG: radiotherapy, olaparib and gemcitabine; PT: proton therapy; PTG: proton therapy and gemcitabine; PTO: proton therapy and olaparib; PTOG: proton therapy, olaparib and gemcitabine.

Comparison (A vs. B)	Number of DE Transcripts	Number of lncRNA
CTL vs. RT	9	1 (11.1%)
RTG vs. RT	150	10 (6.67%)
PT vs. RT	417	29 (6.95%)
RTO vs. RT	0	0 (0%)
RTOG vs. RT	266	17 (6.39%)
RTG vs. RTOG	39	1 (2.6%)
RTO vs. RTOG	38	4 (10.5%)
CTL vs. PT	778	46 (5.91%)
PT vs. PTG	128	9 (7.03%)
PTO vs. PT	657	8 (1.2%)
PTOG vs. PT	1679	61 (3.63%)
PTG vs. PTOG	0	0 (0%)
PTO vs. PTOG	44	6 (13.63%)
RTOG vs. PTOG	0	0 (0%)
PTO vs. RTO	33	1 (0.03%)
PTG vs. RTG	306	20 (6.53%)

## Data Availability

Data are available on GEO.

## Supplementary Materials

The following are available online at https://www.mdpi.com/2072-6694/13/3/527/s1, Figure S1: Kaplan-Meier curve analysis of overall survival according to AURKA expression based on TCGA RNA sequencing data extracted from Genomics Analysis and Visualization Platform (http://r2.amc.nl), Table S1: Median progression-free survival (days) obtained for each condition observed until 50 (photon) and 80 days (proton).
