# Supplementary material for: Radiosensitizing Pancreatic Cancer with PARP Inhibitor and Gemcitabine: An In Vivo and a Whole-Transcriptome Analysis after Proton or Photon Irradiation
Source: Cancers (Basel). 2021 Jan 30;13(3):527. doi: 10.3390/cancers13030527 (PMC7866541; doi:10.3390/cancers13030527)
Supplement: Supplementary file 1 [file cancers-13-00527-s001.pdf]

Supplementary Materials: Radiosensitizing Pancreatic Cancer with PARP Inhibitor and Gemcitabine: An In Vivo and a Whole-Transcriptome Analysis after Proton or Photon Irradiation

Waisse Waissi, Anaïs Nicol, Matthieu Jung, Marc Rousseau, Delphine Jarnet, Georges Noel and Hélène Burckel

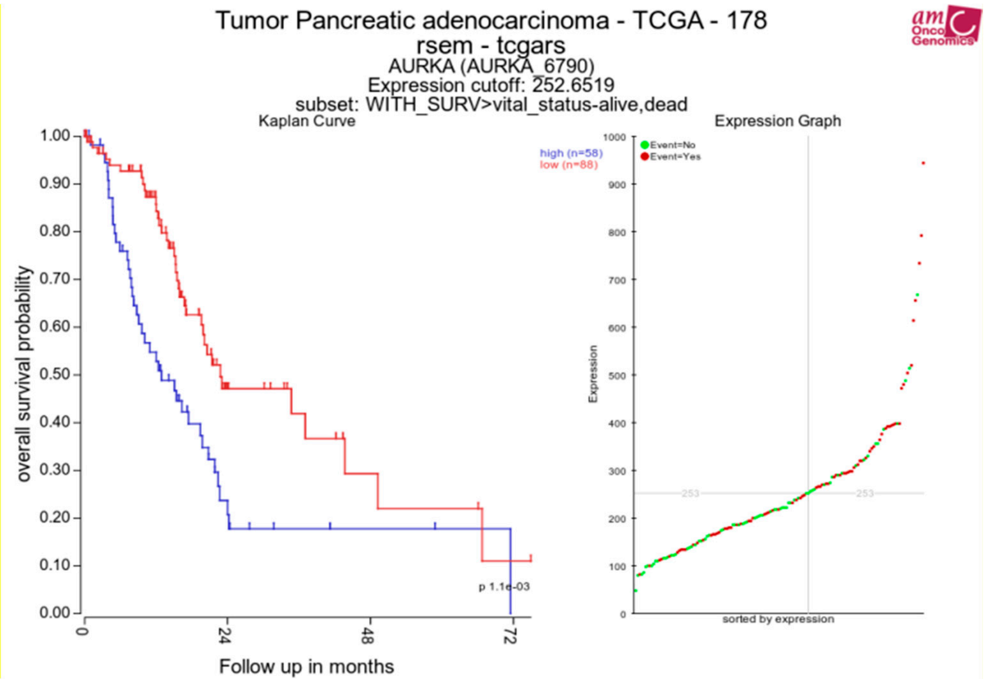

Figure S1. Kaplan-Meier curve analysis of overall survival according to *AURKA* expression based on TCGA RNA sequencing data extracted from Genomics Analysis and Visualization Platform (http://r2.amc.nl).

Table S1. Median progression-free survival (days) obtained for each condition observed until 50 days (photon) and 80 days (proton). DMSO (control); GEM: gemcitabine; OLA: olaparib; NI: not irradiated; NR: never reached.

Irradiation	Systemic Treatment	Median 2 fold Change in Tumor Volume (Days)
NI	DMSO	19 (14–24)
	GEM	19 (19–28)
	OLA	14 (9–28)
	OLA + GEM	23 (16–40)
10 Gy Photon	DMSO	30 (23–NR)
	GEM	37 (30–44)

Copyright: © 2021 by the authors. Licensee MDPI, Basel, Switzerland. This article is an open access article distributed under the terms and conditions of the Creative Commons Attribution (CC BY) license (http://creativecommons.org/licenses/by/4.0/).

	OLA	35 (19–NR)
	OLA + GEM	47 (30–NR)
10 Gy Proton	DMSO	40 (26–NR)
	GEM	77 (44–NR)
	OLA	75 (49–NR)
	OLA + GEM	NR (80–NR)
